# Simulation of acquisition shifts in T2 weighted fluid-attenuated inversion recovery magnetic resonance images to stress test artificial intelligence segmentation networks

**DOI:** 10.1117/1.JMI.11.2.024013

**Published:** 2024-04-24

**Authors:** Christiane Posselt, Mehmet Yigit Avci, Mehmet Yigitsoy, Patrick Schuenke, Christoph Kolbitsch, Tobias Schaeffter, Stefanie Remmele

**Affiliations:** a University of Applied Sciences, Faculty of Electrical and Industrial Engineering, Landshut, Germany; b deepc GmbH, Munich, Germany; c Physikalisch‐Technische Bundesanstalt (PTB), Braunschweig and Berlin, Germany; d Technical University of Berlin, Department of Medical Engineering, Berlin, Germany

**Keywords:** magnetic resonance image simulation, artificial intelligence validation, magnetic resonance imaging sequence, multiple sclerosis lesion segmentation, T2w fluid attenuated inversion recovery

## Abstract

**Purpose:**

To provide a simulation framework for routine neuroimaging test data, which allows for “stress testing” of deep segmentation networks against acquisition shifts that commonly occur in clinical practice for T2 weighted (T2w) fluid-attenuated inversion recovery magnetic resonance imaging protocols.

**Approach:**

The approach simulates “acquisition shift derivatives” of MR images based on MR signal equations. Experiments comprise the validation of the simulated images by real MR scans and example stress tests on state-of-the-art multiple sclerosis lesion segmentation networks to explore a generic model function to describe the F1 score in dependence of the contrast-affecting sequence parameters echo time (TE) and inversion time (TI).

**Results:**

The differences between real and simulated images range up to 19% in gray and white matter for extreme parameter settings. For the segmentation networks under test, the F1 score dependency on TE and TI can be well described by quadratic model functions ($R^{2}>0.9$). The coefficients of the model functions indicate that changes of TE have more influence on the model performance than TI.

**Conclusions:**

We show that these deviations are in the range of values as may be caused by erroneous or individual differences in relaxation times as described by literature. The coefficients of the F1 model function allow for a quantitative comparison of the influences of TE and TI. Limitations arise mainly from tissues with a low baseline signal (like cerebrospinal fluid) and when the protocol contains contrast-affecting measures that cannot be modeled due to missing information in the DICOM header.

## 1 Introduction

In recent years, several machine learning and deep learning (DL) technologies have passed the approval process for a medical device to support radiologists in the diagnosis of medical images.^1^ Still, the reliability of these new medical software devices and the underlying DL networks strongly depends on the training data and how well they represent the variety of real clinical image data (test images). Castro et al.^2^ described different sources of “shifts” between training and test domains and among those, the “acquisition shift, resulting from the use of different scanners or imaging protocols, which is one of the most notorious and well-studied sources of dataset shift in medical imaging.” This is a well-known challenge ever since researchers try to derive reproducible measurements of physiologic information based on heterogeneous medical image data, e.g., by image harmonization in Radiomics research.^3^ In recent years, various studies have been dedicated to minimizing these shifts by domain adaptation methods. For instance, differences between a target and a source domain can be reduced by image preprocessing (e.g., normalizing intensities, or aligning images), by fine-tuning models on target domain data, or by translation of source into target domain images using generative adversarial networks (GANs) or transformers.^4^ These methods have been shown to improve the robustness of artificial intelligence (AI) models but do not provide means for systematic testing and quantification of potential (residual) risks during application. Accordingly, several institutions underline the need for test procedures and published concepts for the evaluation of the robustness and transferability of a model to other data domains.^5^^–^^8^ The ECLAIR guidelines,^8^ for example, request “to check robustness to variability of acquisition parameters.” This is especially important for magnetic resonance imaging (MRI), because MR acquisition protocols typically have a large number of sequence parameters, which affect the contrast, resolution, and SNR of the acquired images. On the one hand, this allows a wide range of clinical information to be presented by MR images, but on the other hand, it leads to a high heterogeneity between different radiology centers. MR acquisition protocols are often optimized individually at each site and sometimes even for different patients to take patient-specific features (e.g. weight and size) into account.^9^ Thus, acquisition parameters may vary even for the same type of scan, hence resulting in different image contrasts. There are guidelines providing recommendations on appropriate MR protocols. Among those, e.g., the recently published MAGNIMS–CMSC–NAIMS consensus guidelines^10^ prescribe the contrast weighting [i.e., T2w, T2w fluid-attenuated inversion recovery (FLAIR), and contrast-enhanced T1w] of the scans that need to be included in the “recommended core” of protocols for the examination of patients with multiple sclerosis (MS). Nevertheless, they lack specific information on contrast-affecting parameters, such as echo, repetition, and inversion time (TE, TR, and TI).

A multitude of visualization methods have been developed to identify the features within images that a neural network is most sensitive to.^11^ Other methods quantify the uncertainty of a network during image processing.^12^ However, there is no test procedure that predicts whether an AI product can be applied to the images of a particular radiology practice without loss of performance, e.g., given their customized imaging protocols. Further, it is currently not possible to determine which acquisition parameters can be changed without compromising the performance of an AI product.

The identification of the influencing factors that a system is most prone to is a well-known problem in the field of process improvement and quality management. It is generally solved by systematic testing based on the “design of experiment (DoE)” concept. DoE is a standardized statistical tool for quality control in Six Sigma processes to systematically evaluate the robustness of a process to its influencing factors (see Ref. 13, Chapter 5.4). It predicts the minimum number of experiments needed to quantify and compare the impact of all influencing factors and their interactions on a system’s outcome or performance metric. Combined with dedicated analysis of the results, the dominating factors can be easily identified. However, to optimize the experimental design to the given problem, regression analysis needs to be performed to estimate the underlying model function that quantifies the dependence of the response variable (here: AI network performance) on the process’ input (here: acquisition parameters), see Ref. 13, Chapter 5.3.3.6.

Therefore, the foremost objective of this work is to study the dependency of a network to the most relevant contrast-affecting acquisition parameters. In the above-mentioned neuroimaging T2w FLAIR scans for example, the TE and the TI have the strongest influence on the imaging contrast. But how can models be validated against the typical MR protocol variability of routine scans or even stress tested against rare but realistic maximum domain shifts if the related data are not available?

The benchmark dataset CLEVR-XAI aims to create a selective, controlled, and realistic test environment for the evaluation of explainable neural networks in non-medical applications.^14^ Similar projects for medical applications have just started.^15^ Using machine learning and neural networks for the simulation and synthesis of medical images is a field of intense research. Attempts have already been made to recreate MRI images through simulation and synthesis, e.g., using GANs or variational autoencoders (VAEs), phantoms, and dedicated multi-parametric MR sequences.^16^ Other simulators use virtual phantoms, for example from Brainweb and Shepp–Logan, which represent the human brain^17^^,^^18^ to generate images that represent a particular protocol. The limiting factors in all the above-mentioned approaches, however, are either the limited number of anatomies (Brainweb), the lack of anatomical realism (Shepp-Logan), the dependency on specific software (sequences), hardware (phantoms), or the ability to synthesize the result of arbitrary MRI sequences settings with only one model (GANs, etc.).

The secondary objective of this study is thus the combination of simulation and synthesis to generate artificial MRI data of arbitrary sequence character (i.e., “shift derivatives”) from a set of real MR images. These data are finally used to stress test a model against variations of acquisition parameters.

For the sake of simplicity, the experiments in this study are focusing on the simulation of domain shift derivatives of T2w FLAIR scans for different TE and TI values to describe the performance of MS lesion segmentation networks in dependence of these scan parameters.

## 2 Methods

This work comprises two levels of methodology and experiments (see Table 1). First, the simulation of domain shift derivatives given a real baseline image dataset, and second, the use of these data to stress test state-of-the-art (SOTA) MS lesion segmentation networks against these shifts. Those networks are trained on data (Table 2) of heterogeneous contrast (e.g., from different field strengths and using different acquisition protocols). The stress tests intend to evaluate to what extent the networks are robust to changes of image contrast. The simulated data are validated by real MRI scans. The dependency of the models’ performance (F1-score) against changes of the MRI protocol parameters (TI, TE) is modeled by second-order polynomial functions, recommended by the above-mentioned DoE guidelines to quantitatively compare the robustness of the networks against acquisition shifts, by the functions’ coefficients.

**Table 1:** Research questions, methodology, and experiments.

Research questions	General methodology	Experiments
How well can acquisition shift derivatives of a (real) MRI dataset be modeled?	1. Estimation of tissue properties (tissue segmentation, partial volume tissue fractions PV, relaxation parameters ${\overset{\to }{p}}_{\text{Relax}}$, texture map $S_{\mathrm{Tex}}$).	1:1 Comparison of simulated and real MRI scans in healthy volunteers by average MR signal values in gray matter, white matter, and CSF. Comparison of the heuristic estimates of T1 and T2 in tissue ROIs with those of relaxometry methods and literature.
	2. Simulation of acquisition shifts based on MRI signal equation dependent on arbitrary sequence parameters ${\overset{\to }{p}}_{\mathrm{Seq}}=(\mathrm{TI},\mathrm{TE})$.	
Is a quadratic model function appropriate to describe the dependence of the F1 score of a segmentation network to acquisition shifts (i.e., changing sequence parameters)?	1. Generation of representative shift derivatives of a real MS dataset.	Modeling of $F1({\overset{\to }{p}}_{\mathrm{Seq}})$ as a second order polynomial function, using $R^{2}$ as a metric to evaluate the model fit.
	2. Measurement of F1 as a function of ${\overset{\to }{p}}_{\mathrm{Seq}}=(\mathrm{TI},\mathrm{TE},)$ in model tests.	

**Table 2:** Datasets used in this work. The first dataset (OpenMS* longitudinal) is utilized as baseline data in the simulation, since this is the only dataset, for which all contrast-affecting parameters (TE, TI, and TR) are provided.

	Data	Nr	Description	Source
**Baseline and Test Data**	OpenMS* (longitudinal)^19^	20	2D FLAIR image: TR = 11000 ms, TE = 140 ms, TI = 2800 ms, FA = 90 deg, sampling: $0.9\times 0.9\times 3\;{\mathrm{mm}}^{3}$	1.5 T Philips, University Medical Centre Ljubljana (UMCL)
**Training Data** ^a^	OpenMS (cross-sectional)^20^	30	3D FLAIR image: TR = 5000 ms, TE = 392 ms, TI = 1800 ms, FA = 120 deg, sampling: $0.47\times 0.47\times 0.80\;{\mathrm{mm}}^{3}$	3 T Siemens Magnetom Trio, University Medical Center Ljubljana
	Lesion challenge 2015^21^	5	2D FLAIR image: TI = 835 ms, TE = 68 ms, sampling: $0.82\times 0.82\times 2.2\;{\mathrm{mm}}^{3}$	3 T Philips, Best, The Netherlands
	Lesion segmentation challenge 2008^22^	20	2D FLAIR image: sampling: $0.5\times 0.5\times 0.5\;{\mathrm{mm}}^{3}$	3 T Siemens
	MSSEG-2^23^	40	3D FLAIR image	1.5 T and 3 T GE, Philips, Siemens
	NAMIC^24^	4	3D FLAIR image: sampling: $1\times 1\times 1\;{\mathrm{mm}}^{3}$	3 T Siemens Magnetom Trio

a The data were split up randomly into 80% and 20% fractions for training and validation of the networks. See Section 2.2.3 for more detail.

The MS data used in this study consist of several open MRI benchmark datasets (see Table 2).

### 2.1 Concept of Image Generation to Mimic Acquisition Shifts

**Fig. 1:**
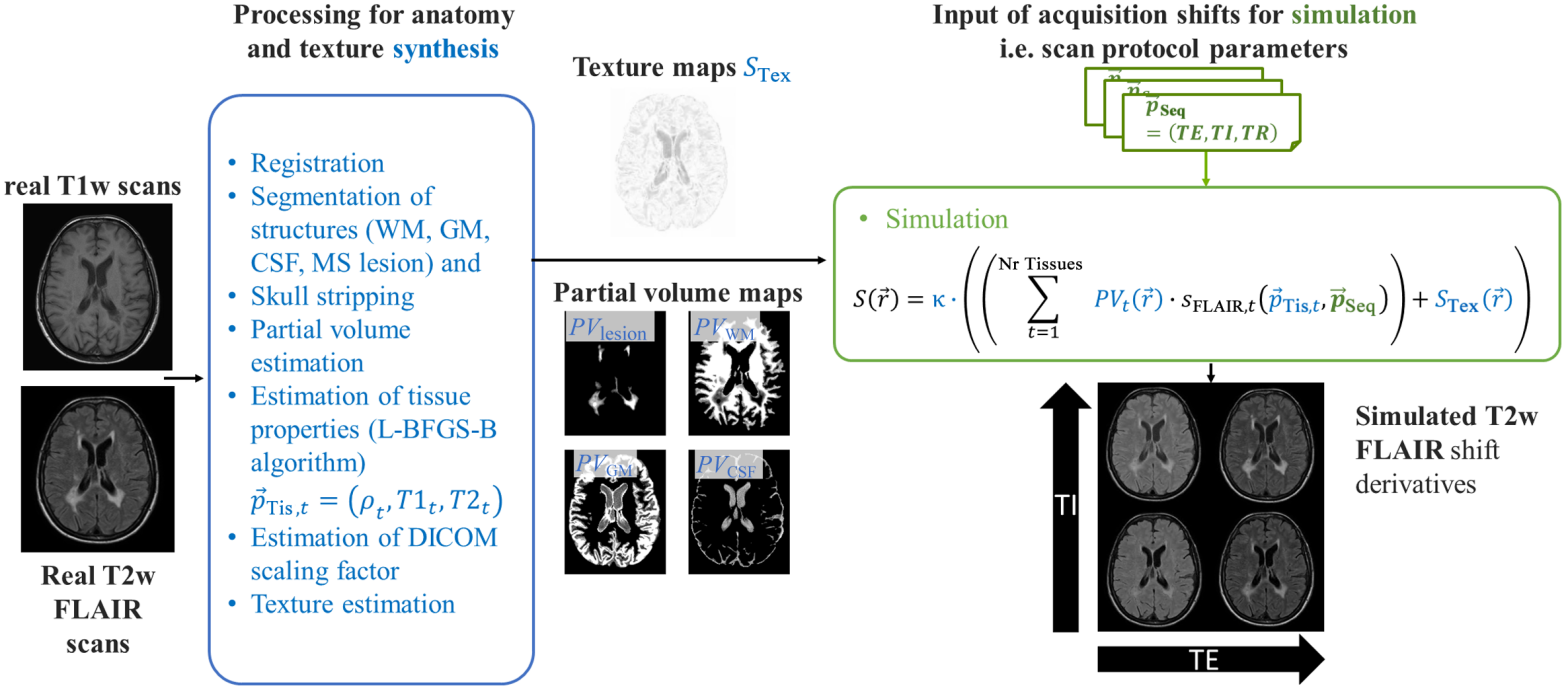
Acquisition shifts of a real baseline dataset are simulated based on the MRI signal equation of a T2w FLAIR sequence. The signal contribution of each tissue t is scaled by its volume fraction $PV_{t}$ and enriched by a texture map $S_{\mathrm{Tex}}$. All influences other than those of the sequence (anatomic structures, DICOM scaling or texture) are synthesized (blue box) from the real baseline scan prior to simulation (green box).

Data simulation uses an *in-vivo* MRI scan (baseline data) and mimics changes in that baseline scan in response to changing sequence parameters. The concept of image generation is based on the following equation:

$$S(\overset{\to }{r})=\kappa \cdot ((\overset{Nr\;\text{Tissues}}{\underset{t=1}{\sum }}PV_{t}(\overset{\to }{r})\cdot s_{\mathrm{FLAIR},t}({\overset{\to }{p}}_{\mathrm{Tis},t},{\overset{\to }{p}}_{\mathrm{Seq}}))+S_{\mathrm{Tex}}(\overset{\to }{r}))\;=((\overset{Nr\;\text{Tissues}}{\underset{t=1}{\sum }}PV_{t}(\overset{\to }{r})\cdot \kappa \cdot s_{\mathrm{FLAIR},t}({\overset{\to }{p}}_{\mathrm{Tis},t},{\overset{\to }{p}}_{\mathrm{Seq}}))+\kappa \cdot S_{\mathrm{Tex}}(\overset{\to }{r})),$$ (1)

with $S(\overset{\to }{r})$, being the simulated signal at pixel position $\overset{\to }{r}=(x,y,z)$. The contribution $s_{\mathrm{FLAIR},t}$ of each tissue t to the signal of a pixel or voxel is weighted with its local volume fraction $PV_{t}(\overset{\to }{r})$. κ is the (typically unknown) digital imaging and communications in medicine (DICOM) scaling factor. The texture map $S_{\mathrm{Tex}}(\overset{\to }{r})$ is introduced to approximate all texture influences other than tissue, e.g., based on artifacts, field inhomogeneities, noise, etc. The entire image generation process therefore consists of two different steps (Fig. 1). The first step comprises the preliminary estimation of these tissue properties followed by the second step, the final image simulation according to Eq. (1).

$s_{\mathrm{FLAIR},t}({\overset{\to }{p}}_{\mathrm{Tis},t},{\overset{\to }{p}}_{\mathrm{Seq}})$ is the signal as determined by the sequence and the tissue properties, i.e., the parameters ${\overset{\to }{p}}_{\mathrm{Tis},t}=(\rho_{t},T1_{t},T2_{t})$ of the underlying tissue t [like the spin density ρ and relaxation parameters T1 and T2 of gray matter (GM), white matter (WM), cerebrospinal fluid (CSF), and lesion]. $s_{\mathrm{FLAIR},t}$ is given by the T2w FLAIR signal equation in Eq. (2) as published in Ref. 25

$$s_{FLAIR,t}({\overset{\to }{p}}_{Tis,t},{\overset{\to }{p}}_{Seq})=\rho_{t}\cdot (1-2\cdot \exp(-\frac{TI}{T1_{t}})+\exp(-\frac{\left(TR-TE_{\text{last}}\right)}{T1_{t}}))\cdot \exp(-\frac{TE}{T2_{t}})$$ (2)

with ${\overset{\to }{p}}_{\mathrm{Seq}}=(\mathrm{TE},\mathrm{TI},\mathrm{TR},\ldots )$, i.e., the sequence parameters.

#### 2.1.1 Simulation and synthesis methods

Equations (1) and (2) contain a number of tissue parameters that must be represented as realistic as possible for the data generation process but cannot be easily simulated (e.g., anatomical structures, lesion sizes, and locations). The idea behind the proposed generative approach is thus to combine image synthesis and simulation as follows.

1. Synthesis: anatomy and disease related signal contributions are derived from a real MR baseline dataset $S_{m}$. These data are used to estimate:
  • the partial volume maps $PV_{t}(\overset{\to }{r})$ using a partial volume estimation method based on Ref. 26 (see next section). The approach requires an additional T1w scan, which is also included in the above-mentioned “recommended core” protocols of MS examinations. The approach further requires prior tissue segmentation.
  • the DICOM scaling factor κ of the baseline T2w FLAIR scan and
  • $S_{\mathrm{Tex}}(\overset{\to }{r})$, to mimic other texture influences (e.g., noise and artifacts).
2. Simulation of all signal contributions that are affected by the sequence and the choice of parameters.
  • Simulation of acquisition shifts is performed through variation of ${\overset{\to }{p}}_{\mathrm{Seq}}$ in $s_{\mathrm{FLAIR},t}({\overset{\to }{p}}_{\mathrm{Tis},t},{\overset{\to }{p}}_{\mathrm{Seq}})$ using Eq. (2). T1 and T2 are set to random values within a realistic range.

#### 2.1.2 Partial volume estimation

**Fig. 2:**
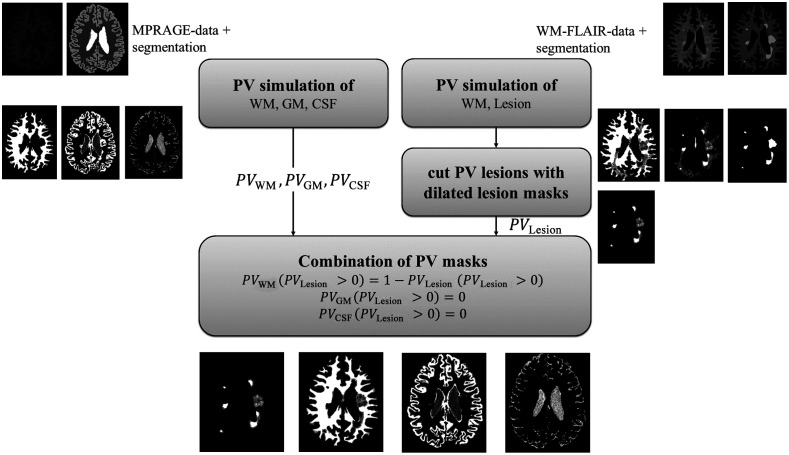
Partial volume (PV) maps for normal tissue are determined based on a T1w scan and the method described in Ref. 26. The PV map for the lesion is estimated using the same method and a WM-lesion segment of the T2w FLAIR scan, where lesions differentiate better from the WM background. Fusing all PV information yields the final PV maps.

For estimation of the partial volume fractions of each tissue, we apply the method described in Ref. 26. This approach requires that a signal rise or decline from one region to the other is unique for one kind of tissue-tissue interface. However, in case the brain contains lesions, a rise of signal when leaving the WM region may be attributed to either a WM-lesion or a WM-GM interface. The partial volume maps are thus generated in two steps, assuming that lesions are solely located in and surrounded by WM.^27^ First, as required by the approach, segmentation masks are created. We used Synthseg^28^ for segmentation of normal tissues, and expert lesion masks were provided through the datasets.^29^ Second, the T1w scans are used to estimate the PV-maps ${\mathrm{PV}}_{\mathrm{WM}1}$, ${\mathrm{PV}}_{\mathrm{GM}}$, and ${\mathrm{PV}}_{\mathrm{CSF}}$ of normal tissue. Lesion pixels might be falsely assigned to the PV-map of GM, which can be easily corrected by setting the GM maps to 0 at all lesion pixels as given by segmentation. Third, WM and lesion ROIs are extracted from the FLAIR images and are fed through the PV-algorithm, to obtain another ${\mathrm{PV}}_{\mathrm{WM}2}$ and ${\mathrm{PV}}_{\text{lesion}}$ map. The final ${\mathrm{PV}}_{\mathrm{WM}}$ is initialized with ${\mathrm{PV}}_{\mathrm{WM}1}$. Finally, in pixels, where ${\mathrm{PV}}_{\text{lesion}}>0$, the partial volume fraction in WM is then set to ${\mathrm{PV}}_{\mathrm{WM}}=1-{\mathrm{PV}}_{\text{lesion}}$. All steps are summarized in Fig. 2.

#### 2.1.3 Estimation of the DICOM scaling factor κ and the texture map $S_{\mathrm{Tex}}(\overset{\to }{r})$

A simplified version of Eq. (1) describes the signal of those pixels of the real baseline image $S_{m}$ that contain only one tissue fraction (PV=1)

$$S_{m,t}(\overset{\to }{r})=\underset{\text{Signal term}}{\underset{⏟}{\kappa \cdot 1\cdot s_{\mathrm{FLAIR},t}({\overset{\to }{p}}_{\mathrm{Tis},t},{\overset{\to }{p}}_{\mathrm{Seq}})}}+\underset{\text{Texture term}}{\underset{⏟}{\kappa \cdot S_{\mathrm{Tex}}(\overset{\to }{r})}}.\;\text{for all}\;{\overset{\to }{r}}_{PVt=1},\;\text{where}\;PV_{t}(\overset{\to }{r})=1.$$ (3)

Since both $S_{\mathrm{Tex}}$ and ${\overset{\to }{p}}_{\mathrm{Tis},t}$ are unknown, the problem of computing $S_{\mathrm{Tex}}$ is overdetermined. We solve this by introducing the assumption that signal variations are primarily caused by noise and thus the average texture ${\overset{¯}{S}}_{\mathrm{Tex}}({\overset{\to }{r}}_{\mathrm{PV}t=1})$ in this region is 0. Eq. (3) can then be written as

$${\overset{¯}{S}}_{m,t}({\overset{\to }{r}}_{\mathrm{PV}t=1})=\kappa \cdot s_{\mathrm{FLAIR},t}({\overset{\to }{p}}_{\mathrm{Tis},t},{\overset{\to }{p}}_{\mathrm{Seq}}).$$ (4)

This allows for a preliminary estimation of the apparent tissue parameters ${\overset{˜}{\overset{\to }{p}}}_{\mathrm{Tis},t}$ from the ratio of average real and simulated signals for different tissues t [the ratio eliminates the unknown κ in Eq. (4)], or more precisely by comparing the real and simulated contrast metrics given in the following equations:

$$C_{\text{sim},t_{1},t_{2}}=\frac{s_{\text{FLAIR},t_{1}}({\overset{\to }{p}}_{\text{TisEst},t_{1}},{\overset{\to }{p}}_{\text{Seq}})-s_{\text{FLAIR},t_{2}}({\overset{\to }{p}}_{\text{TisEst},t_{2}},{\overset{\to }{p}}_{\mathrm{Seq}})}{s_{\text{FLAIR},t_{1}}({\overset{\to }{p}}_{\text{TisEst},t_{1}},{\overset{\to }{p}}_{\text{Seq}})+s_{\text{FLAIR},t_{2}}({\overset{\to }{p}}_{\text{TisEst},t_{2}},{\overset{\to }{p}}_{\mathrm{Seq}})},$$ (5)

$$C_{m,t_{1},t_{2}}=\frac{{\overset{¯}{S}}_{m,t_{1}}({\overset{\to }{r}}_{{PVt}_{1}=1})-{\overset{¯}{S}}_{m,t_{2}}({\overset{\to }{r}}_{{PVt}_{2}=1})}{{\overset{¯}{S}}_{m,t_{1}}({\overset{\to }{r}}_{{PVt}_{1}=1})+{\overset{¯}{S}}_{m,t_{2}}({\overset{\to }{r}}_{{PVt}_{2}=1})}.$$ (6)

The parameters of ${\overset{\to }{p}}_{\mathrm{Tis},t}$ are optimized to minimize the cost function

$$(C_{\mathrm{sim},\mathrm{GM},\mathrm{WM}}-C_{m,\text{GM},\text{WM}}{)}^{2}+(C_{\text{sim},\text{CSF},\text{WM}}-C_{m,\text{CSF},\text{WM}}{)}^{2}\;+{\left(C_{\mathrm{sim},\text{Lesion},\mathrm{WM}}-C_{m,\text{Lesion},\text{WM}}\right)}^{2}\to \text{min}.$$ (7)

**Fig. 3:**
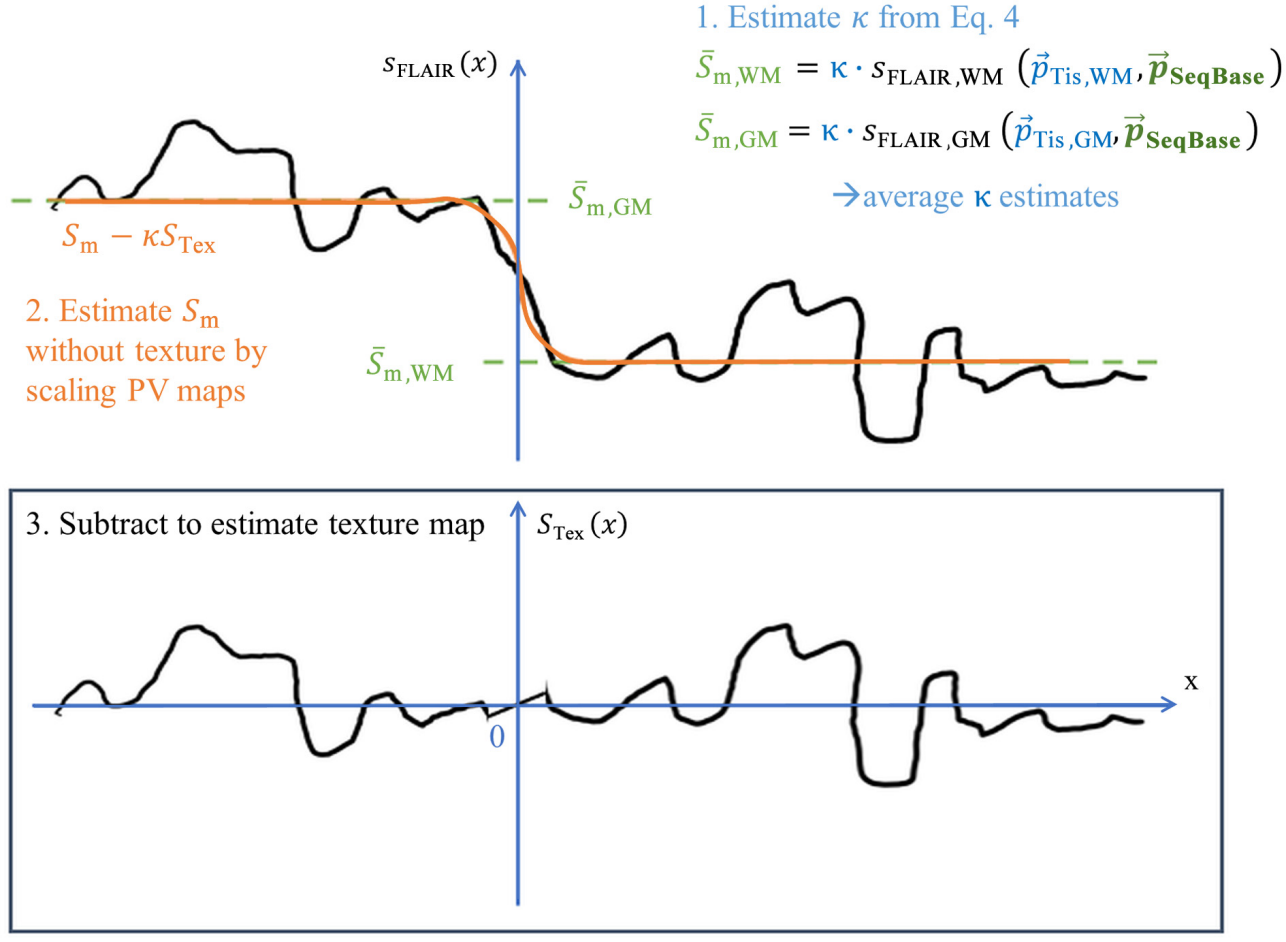
Method to estimate the texture of an MR image by subtracting the estimated signal in consideration of the partial volume effect.

Then, κ can be estimated using Eq. (4). Now, that all unknowns are determined, Eq. (1) is solved to determine the texture map $S_{\mathrm{Tex}}(\overset{\to }{r})$ (see Fig. 3).

#### 2.1.4 Experiments - comparison of simulation and measurement

MR images of 10 healthy volunteers were acquired to compare the simulations with real measurements. The examinations were approved by the ethics committee of the Physikalisch-Technische Bundesanstalt and are in accordance with the relevant guidelines and regulations. Written informed consent was obtained from all volunteers prior to the measurements. Data were acquired at 3T (Siemens Verio) using the following sequences: a magnetization prepared rapid gradient echo for the estimation of the PV-maps (3D, TR = 2300 ms, TI = 900 ms, TE = 3.2 ms, voxel size: $0.75\times 0.75\times 4.69\;{\mathrm{mm}}^{3}$) and five T2w FLAIR scans as a reference measurement for the simulated images (Multislice 2D, TR = 9000 ms, voxel size: $0.75\times 0.75\times 4.69\;{\mathrm{mm}}^{3}$) with TE and TI values as given in Table 3 to represent the extreme shift derivatives of the possible scan domain and its center (see Fig. 5). The “center” protocol serves as the baseline scan for the simulations of the “corner” protocols.

**Table 3:** TE and TI of the five T2w FLAIR acquisition protocols.

TE/ms	TI/ms
112	2500
84	2200
84	2900
150	2200
150	2900

Reference T1 values were obtained from saturation-recovery measurements. Eleven T1-weighted images for different saturation delay times (TD = 0.1, 0.2, 0.3, 0.4, 0.5, 0.75, 1.0 1.25, 1.5, 2.0, and 8.0 s) were acquired using a fully sampled single-shot centric-reordered GRE readout (TE/TR = 3.0/6.5 ms, flip angle = 6 deg, voxel size: $1.3\times 1.3\times 8.0\;mm^{3}$) implemented in pulseq.^30^ Final quantitative T1 values were generated using a non-linear least squares curve fitting algorithm^31^ assuming a simple mono-exponential magnetization recovery. T2 reference values were derived from the two different TEs (${\mathrm{TE}}_{1}=84\;\mathrm{ms}$ and ${\mathrm{TE}}_{2}=150\;\mathrm{ms}$) of the FLAIR scans $S_{m}$ using the following equation:

$$T_{2}=\frac{{\text{TE}}_{2}-{\text{TE}}_{1}}{ln(\frac{S_{m}({\text{TE}}_{1})}{S_{m}({\text{TE}}_{2})})}.$$ (8)

**Fig. 4:**
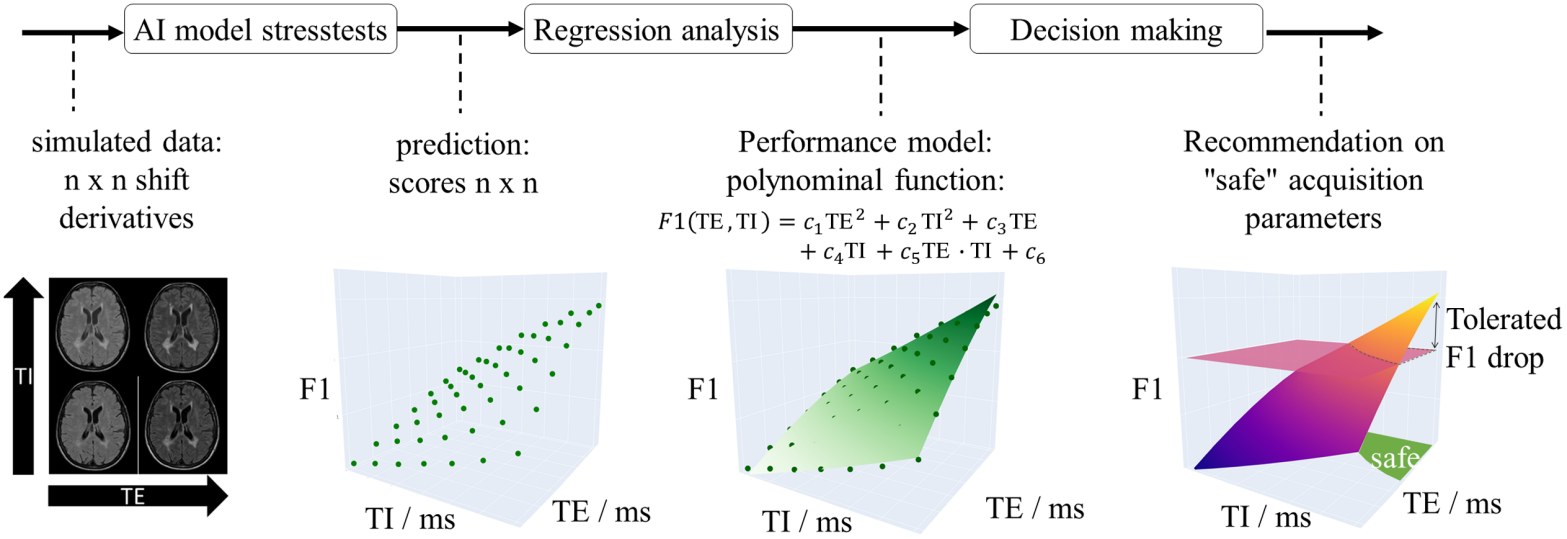
The AI model undergoes testing using generated images that represent varying acquisition shifts. Regression analysis delivers a model function for F1 to provide the user with an assessment of the model’s limitations.

The T2 estimates obtained with TI = 2900 ms and 2200 ms are averaged to deliver the final reference T2 values. The relaxometry estimates described in Sec. 2.1.3 are compared to these reference values and to values given by literature.^32^^–^^35^ Finally, the five real and simulated scans are compared by the theoretical percentage signal deviation per ms relaxometry errors ΔT1 and ΔT2 approximated by error propagation as

$${ds}_{\text{T1}}(\text{T1})=\frac{\frac{{ds}_{\text{FLAIR}}(T1,T2)}{dT1}}{s_{\text{FLAIR}}}\cdot 100\%,\;{ds}_{T2}(T2)=\frac{\frac{{ds}_{\text{FLAIR}}(T1,T2)}{dT2}}{s_{\text{FLAIR}}}\cdot 100\%,$$ (9)

and in dependence of T1 and T2 to confirm that signal differences are related to relaxometry imperfections. The stress test pipeline is summarized in Fig. 4 and comprises two steps as described in the following sections.

### 2.2 Model Stress Tests to Determine the Influence of Acquisition Shifts

#### 2.2.1 Generation of test data

**Fig. 5:**
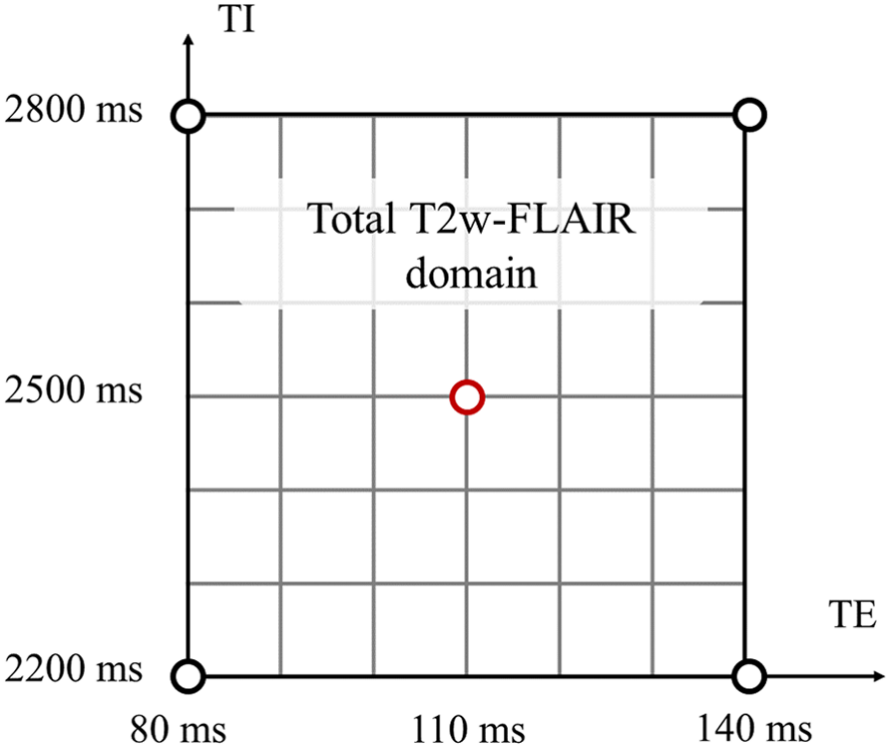
Minimum and maximum values of TI and TE as determined by literature research and real scans. These values limit the real-world scan domain. Test data are generated by simulation to represent all possible data within this domain on a regular grid. The corners and the center (red circle) determine the MRI protocols for reference measurements used to validate the simulated data.

With the methods described in 2.1, derivatives of the baseline data can be generated that represent arbitrary acquisition shifts of a baseline scan (“shift derivatives”). Typical variations of scan protocols (minimum and maximum TE and TI values) were estimated using literature and real scans. The outcome of that investigation is published in Ref. 36 and is depicted in Fig. 5.

7×7 test datasets were generated that represent seven different TE values and seven different TI values, since these are the most contrast-affecting parameters in T2w FLAIR sequences.

#### 2.2.2 Modeling the network performance in dependence of sequence parameters

The lesion F1 score of a lesion segmentation network can be determined for all of these data comparing the network prediction with the lesion ground truth segmentation masks. Averaging all lesion F1 scores finally delivers F1 as a function of TE and TI. We use a response surface method (quadratic model, cubic terms neglected) to describe the dependence of F1 on arbitrary values of the influencing factors TE and TI and their interactions as recommended by Ref. 13. Accordingly, the quadratic model in Eq. (10) is fitted to these F1 measurements

$$F1(\text{TE},\text{TI})=c1\cdot {\text{TE}}^{2}+c2\cdot {\text{TI}}^{2}+c3\cdot (\text{TI}\cdot \text{TE}{)}^{2}+c4\cdot \text{TE}+c5\cdot \text{TI}+c6\cdot \text{TI}\cdot \text{TE}+c7.$$ (10)

The coefficients c1 to c6 can each be understood as a measure of the relevance of the influencing factors TE and TI (main factors) and their interactions TE·TI.

#### 2.2.3 Experiments - stress testing SOTA models against acquisition shifts

To validate the model function described in Eq. (10), two SOTA models are trained on data with heterogeneous contrast as described in Table 2. First, the nnU-Net framework is used, which utilizes a U-Net architecture and automatically configures its hyperparameters and configuration.^37^ The first model is a 3D full-resolution nnU-Net, which is chosen by nnU-Net’s auto-configured framework as the best-performing model among 2D and low-resolution 3D counterparts. Training is done by nnU-Net’s self-configured automatic framework, where fivefold cross-validation is employed with 80% for training and 20% for validation, and the best-performing fold is chosen as the final model. The second model is a SegResNet model, which uses ResNet-like blocks and skip connections without the variational autoencoder part.^38^ The network is trained with 64×64×64 cropped blocks for 1000 epochs with an Adam optimizer and learning rate of 0.001 with Pytorch and MONAI tools. The training data are randomly split into fractions of 80% for training and 20% for validation.

The “longitudinal” OpenMS dataset is the only open benchmark dataset for which all contrast-affecting parameters (TE, TI, TR) are provided (Table 2). All data are skull stripped using the FSL brain extraction tool (FSL BET)^39^ prior to all processing steps. The average F1 is determined and modeled as a function of TE and TI as described in 2.2.2. $R^{2}$ is used to evaluate the appropriateness of the model function in Eq. (10).

## 3 Results

### 3.1 Comparison of Simulation and Measurement

**Fig. 6:**
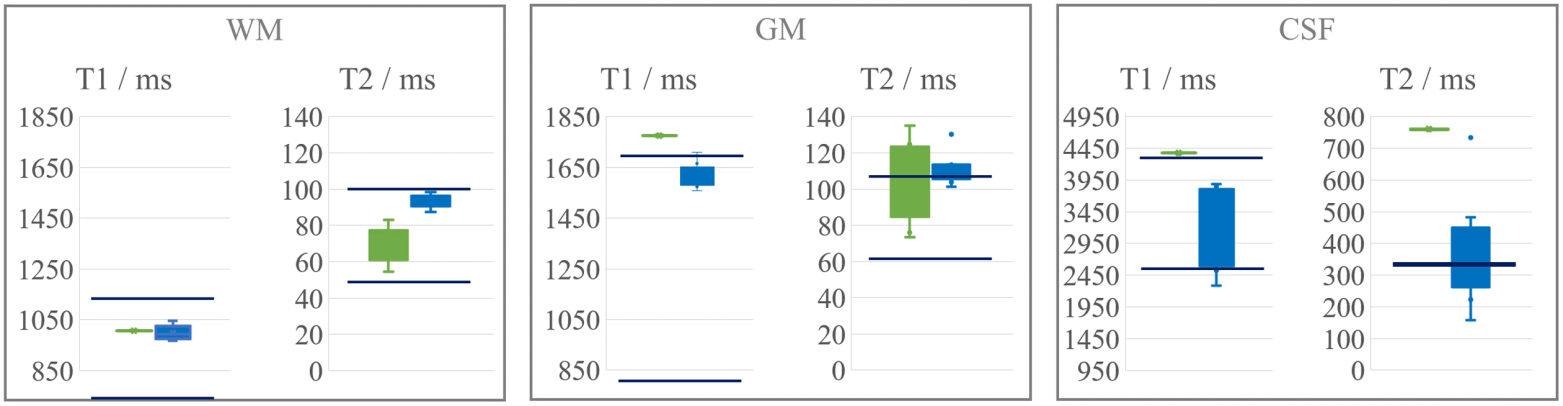
Comparison of the ranges of estimated (green) and reference relaxation measurements (blue) at 3T. Blue lines show the literature ranges.^32^^–^^35^

Figure 6 shows the variation of the estimated and reference relaxation measurements in comparison to the literature ranges. The estimated and measured relaxation times mostly lie within the literature range. As further underlined by the mean relaxometry values in Table 4, the high T1 value and the low FLAIR signal hampers relaxometry in CSF. The literature does not report on CSF T2 measurements at 3T. T2 is independent of the field strength but even at 1.5 T, to our knowledge, the Brainweb catalogue is the only literature source reporting a T2 value for CSF (329 ms), although the values presented in that catalogue (in WM and GM) tend to be lower than most other values at 1.5 T.^40^

**Table 4:** Mean values for T1 and T2 in normal tissue. All values are given in ms.

	$T1_{\mathrm{wm}}$	$T2_{\mathrm{wm}}$	$T1_{\mathrm{gm}}$	$T2_{\mathrm{gm}}$	$T1_{\mathrm{csf}}$	$T2_{\mathrm{csf}}$
**Estimated**	1007 ± 1	69 ± 9	1776 ± 3	102 ± 20	4376 ± 4	760 ± 379
**Measured**	999 ± 27	94 ± 3	1616 ± 46	111 ± 8	3176 ± 568	379 ± 157

**Fig. 7:**
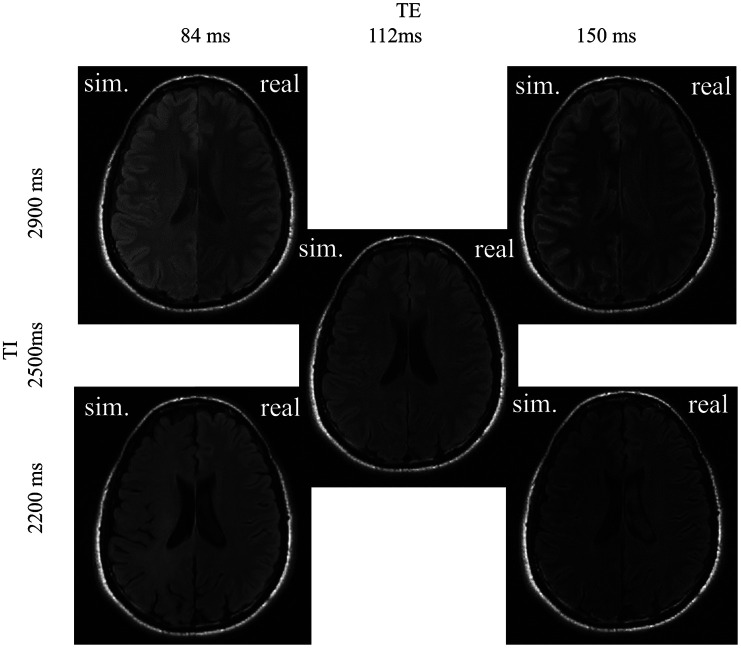
MRI simulations (left side of the MRIs) and their real counterparts (right side of the MRIs) for all five protocols of one example volunteer. The simulation results are embedded in the skull segment to adjust the scaling of the images.

Visually, the images obtained by the simulations and measurements agree well (Fig. 7). Small scaling errors of the nulled CSF signal result in high relative signal deviations. In addition, Table 5 lists the relative error between real and simulated images in different manually drawn ROIs.

**Table 5:** Comparison of the mean signals of WM, GM, CSF, and skull of simulation and reference MRI with relative percentage error.

TE / ms	150	150	112	84	84
TI / ms	2900	2200	2500	2900	2200
WM / %	18	19	0	13	12
	±6	±6	0	±6	±6
GM / %	7	9	0	8	8
	±3	±7	±0	±6	±5
CSF / %	75	36	0	22	58
	±30	±9	±0	±13	±10

**Fig. 8:**
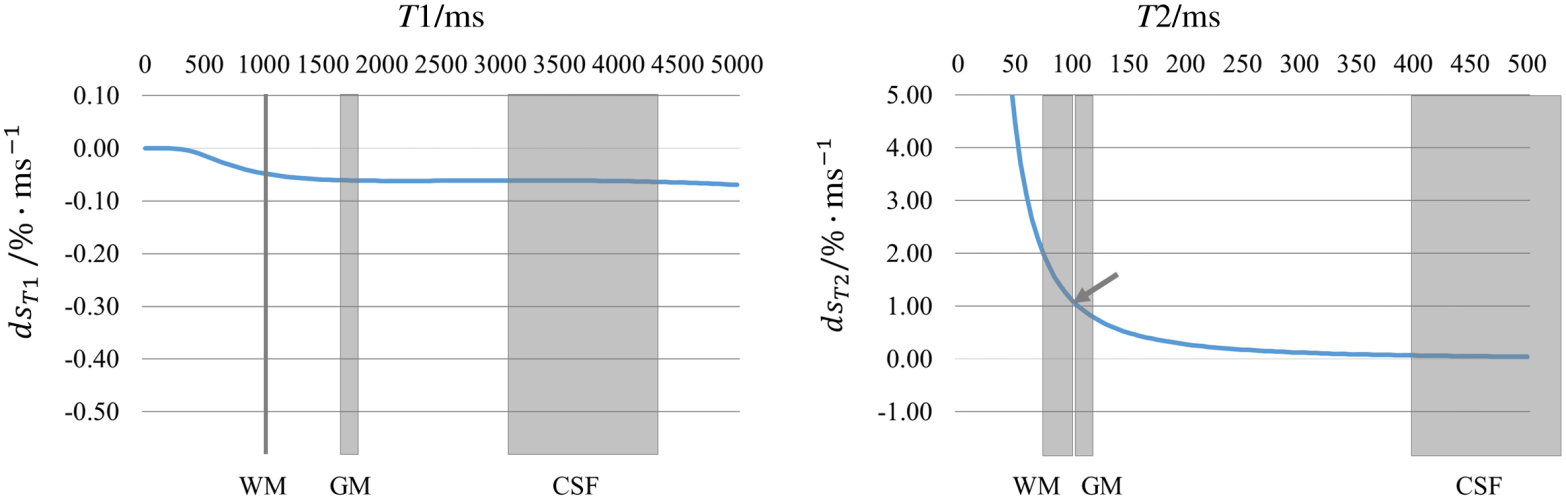
Percentage signal simulation errors per ms relaxometry value as described by error propagation in dependence on the tissue’s T1 or T2 values; e.g., (see arrows) the overestimation of T2 by 1 ms results in about 1% signal simulation error of WM and GM signals (here: given average protocol parameters). The absolute errors increase with T1 and decrease with T2.

The deviation between the simulated and the measured MR signals in WM is higher than in GM. The theoretical error propagation of the relaxometry estimates on the simulated signal is depicted in Fig. 8.

### 3.2 Results of Stress Testing SOTA Models Against Acquisition Shift

**Fig. 9:**
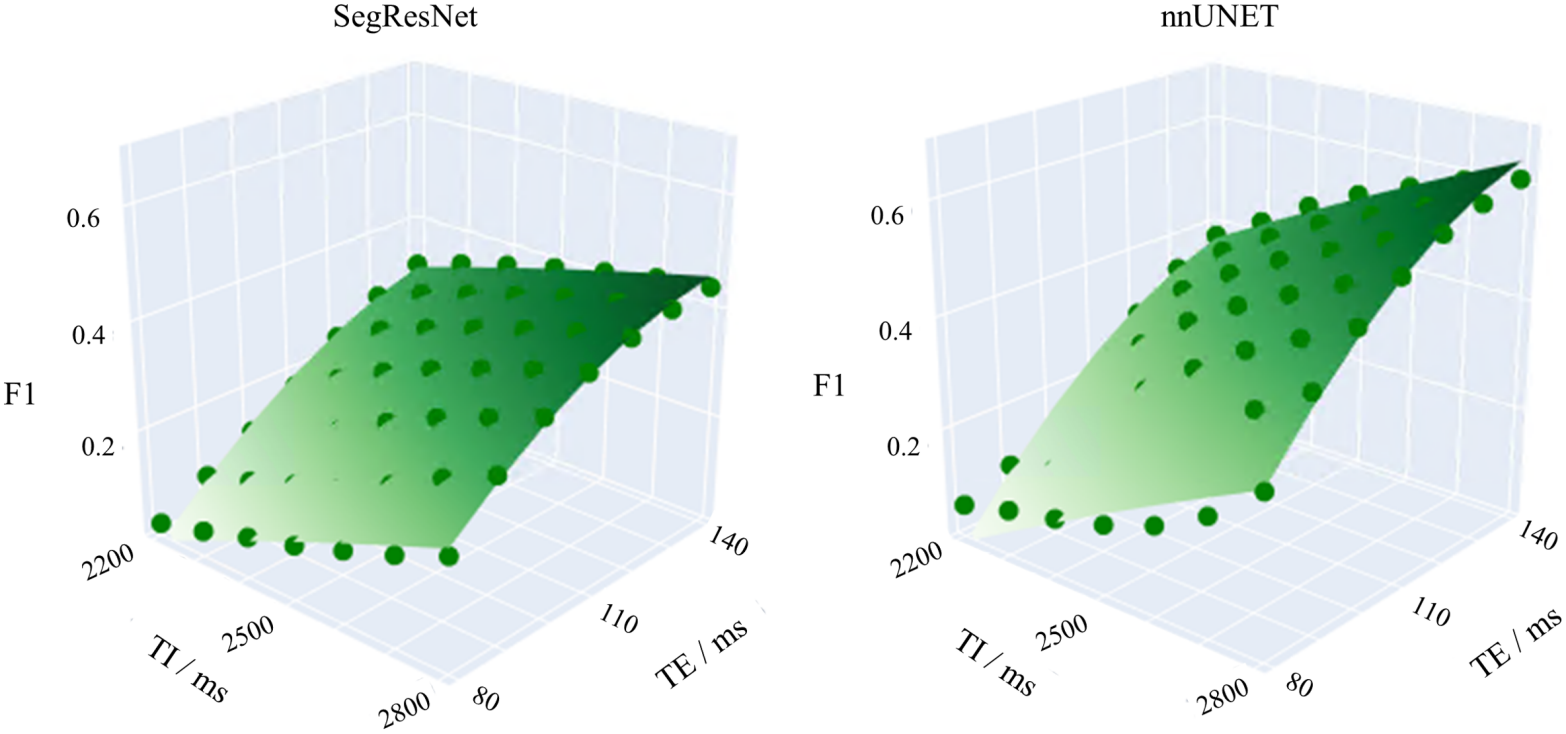
The surface plots show the behavior of the AI models in dependence of the data shifts. Points: F1 scores of the predictions, surface: model fit, i.e., the F1 trend as a function of the acquisition parameters TE and TI.

Testing the models with the real baseline data and their simulated counterpart (TE = 140 ms and TI = 2800 ms) yields F1 scores, which differ in the fourth decimal place (OpenMS data: SegResNet: 0.4398±0.2242; nnU–Net: 0.6105±0.1500, see Fig. 9). The coefficient of determination $R^{2}$ of the model fit (second-order polynomial) is 0.991 for the SegResNet results and 0.982 for the nnU-Net results.

The coefficients for Eq. (10) in Table 6 show that TE has the highest influence on both segmentation networks.

**Table 6:** Coefficients c1 to c7 as given by the model fit (see Eq. 10). Units are given in ${\text{ms}}^{-1}\;$ and ${\text{ms}}^{-2}$ for linear, quadratic, and combined terms, respectively. The highest coefficients are those scaling the influencing factor TE.

	Intersection	TE	TI	${\mathrm{TE}}^{2}$	${\mathrm{TI}}^{2}$	TE·TI
**nnU-Net**	−2.95	$2.24\cdot 10^{-2}$	$9.42\cdot 10^{-4}$	$-6.48\cdot 10^{-5}$	$-7.59\cdot 10^{-8}$	$-8.14\cdot 10^{-7}$
**SegResNet**	−2.57	$2.14\cdot 10^{-2}$	$8.25\cdot 10^{-4}$	$-5.15\cdot 10^{-5}$	$-5.37\cdot 10^{-8}$	$-2.18\cdot 10^{-6}$

**Fig. 10:**
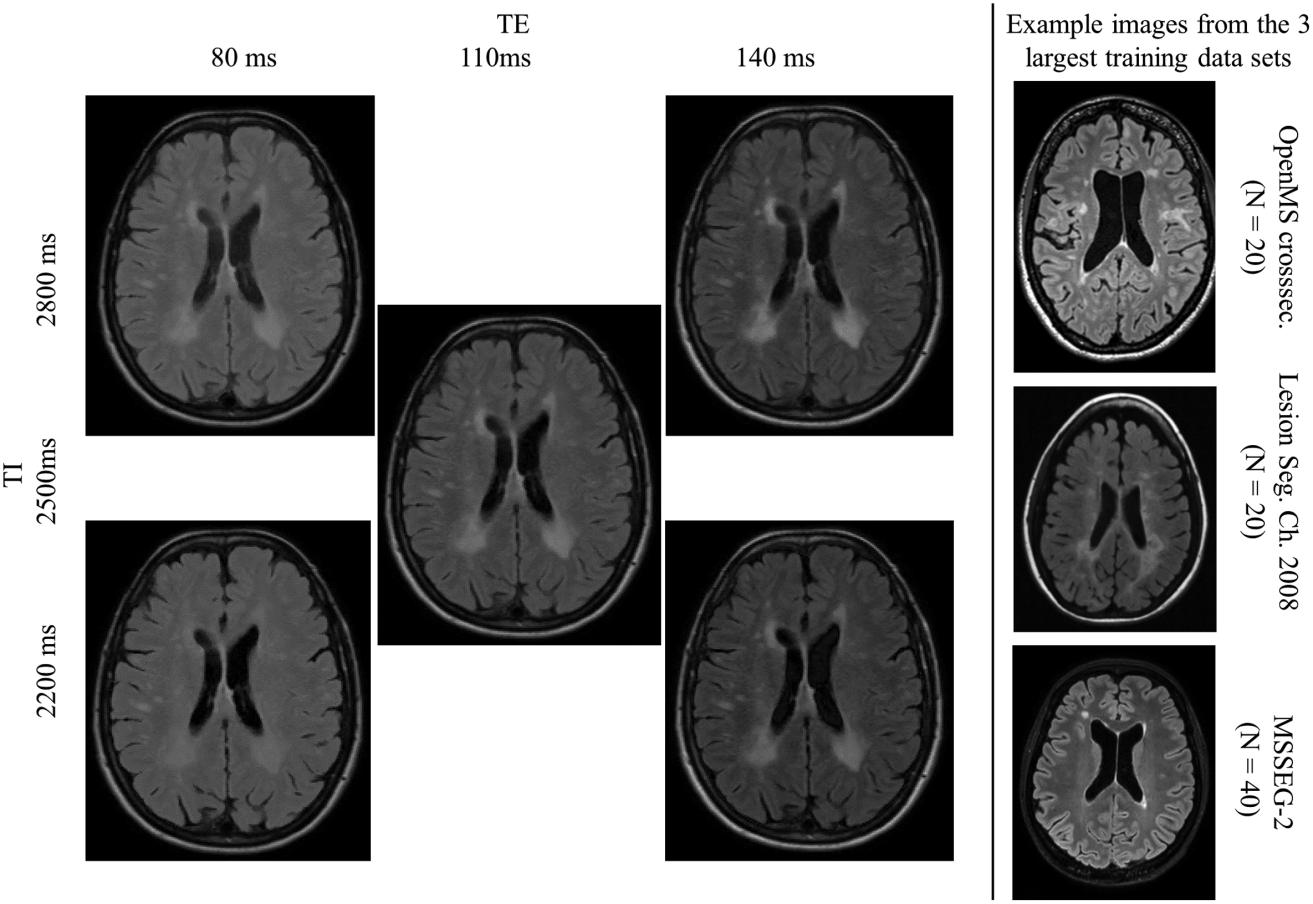
Left: MRI simulations for five protocols (Fig. 5) of one patient of the OpenMS dataset. The simulation results are embedded in the skull segment to adjust the scaling of the images. Right: Example images of the most relevant training datasets. The lesions differentiate well from the WM background, comparable to the shift derivatives with higher lesion-WM contrast (at high TE and TI).

In the simulated images of Fig. 10, the lesion-to-WM contrast decreases for lower TE and TI values. This is accompanied by a decline of the F1-score, i.e., the models’ ability to differentiate between the lesion and white matter decreases with lower contrast.

## 4 Discussion and Conclusion

The image generation method simulates acquisition shift derivatives of a real baseline scan for arbitrary sequence parameters. It was designed to be applicable to common clinical neuroimaging studies that normally contain T2w FLAIR and T1w images. It does not require extra sequences but only knowledge of the scan parameters of the baseline T2w FLAIR data.

### 4.1 Comparison of Simulation and Measurements

At the extreme points of the experimental design, the simulation shows a 19% deviation to the measured values in white matter and lower deviation in gray matter. This can most likely be explained by the inaccuracies of the relaxometry method used in this work. Using the error propagation as a rough guess, the misestimation of 19% could be explained by a 19 ms deviation of T2, which is likely to be realistic considering the reference measurements and the range of literature reference values. Even those reference relaxometry methods suffer from inaccuracies caused by inflow or sequence imperfections, in particular when estimating the T1 and T2 of flowing tissue like blood or CSF.^41^ One could improve the validation by including T1 and T2 mapping sequences in the same resolution and spatial coverage. Common relaxometry sequences in neuroimaging rely on multiple 3D spoiled gradient recalled echo or inversion recovery sequences for T1 mapping and multi-echo or balanced steady-state free precession sequences at variable flip angles for T2 mapping.^41^^,^^42^ The imaging study in this work was already time-consuming due to the five times repetition of the lengthy T2w FLAIR protocol and the T1 weighted scan. Therefore, there was just limited time for a rough dual echo T2 estimation and for the addition of a time-efficient single-slice T1-mapping protocol (acquisition time ∼30  s) to examine the T1 estimates in one slice, and thus values were compared ROI-wise. Still, the T1 and T2 values estimated here mostly lie in the range of literature values, and differences in the reference measurements are also comparable to the range of literature values. A one-to-one comparison of real and simulated images is challenging as it requires the exact knowledge of the relaxation times of that particular patient. Precise relaxometry is neither the aim of this work nor is it necessary for the simulation of test data. The relaxometry parameters in Eqs. (1) and (2) are set to arbitrary values to deliver a representative cohort of anatomies. Relaxometry imperfections hamper accurate validation of the simulated values, yet, they manifest only in a misestimation of the DICOM scaling factor κ and thus in under- or overestimation of the texture amplitude. Unfortunately, for MRI sequences this scaling factor is not part of the DICOM header as it is for the Hounsfield units in CT imaging. Irregularities of the texture amplitude, on the other hand, might be balanced by normalizing the texture amplitude over the entire dataset. Furthermore, the texture amplitude could be also included as another influencing factor in the stress test analysis in addition to the sequence parameters—e.g., as a measure of noise or artifact level. In contrast to using other AI-based generative approaches like GANs, VAEs, or diffusion models,^16^^,^^43^^–^^46^ the underlying signal equation allows for the generation of arbitrary but distinct shift derivatives from just one dataset.

### 4.2 Stress Test Results

The stress test results between the two networks differ, either due to their architectures or different data splits used for training and validation. However, in both cases, the F1(TE, TI) measurements seem to be well described by the quadratic function. The metric varies only smoothly so that cubic terms can be neglected. TE seems to be the most influencing factor for all models, which is in line with the nature of the contrast weighting of the sequence (T2w FLAIR).

Furthermore, the lesion F1 values are comparable to that of real data (72%^47^) at least in or close to the baseline representation. The performance decreases towards the extreme points of the experimental grid (particularly for low TE values), where the lesion-WM contrast decreases. As one can see in Fig. 10 (example training images), the lesion-WM contrast of the training images was generally higher than in the low-TE simulations, which might explain the performance drop towards low TE values. In previous work, using fully simulated data, we showed that the maximum of the response surface plot and its shape are dependent on the contrast distribution of training and test data.^36^ The stress test result can thus be a measure of model analysis and optimization. One has to bear in mind that these extreme points are mathematical constraints, given by the minimum and maximum combinations of TE and TI of real sequences. The boundary of the experimental grid does not represent the boundary of the typical scan domain. The latter does not necessarily contain the combination of extreme values of both TE and TI at the same time. Those extreme data simulations are thus not part of the training data therefore causing severe drops in the F1 value.

The high F1 scores for the two “high-TE corners” (Fig. 9) can also be explained by the high lesion contrast for these protocols. In contrast, the low lesion contrast yielded by low TE and TI values comes with low F1 scores, respectively. Another contribution of this work is thus a proof-of-concept for the description of the performance metric of an AI model in dependence of its influencing factors. The modeling yields a quantitative comparison of the relevance of all influencing factors. This concept of surface response modeling is based on well-established experimental designs and could be easily transferred to other common metrics^48^ (e.g., confusion matrix and derivatives or even uncertainty estimates^49^) or other models (e.g., classification models). Now, that the model function was confirmed, the number of experiments could be reduced significantly in future studies to reduce the computational effort. For the optimal “positioning” of these sample points on the “domain grid” for meaningful sampling of the surface response curve, state-of-the-art guidelines in the field of experimental design offer several recommendations depending on the number of influencing factors.^13^

### 4.3 Limitations

One important limitation is the small number of test datasets used in this study. Thus, the absolute results of the stress tests might not be representative for a larger cohort of patients and lesions. They can only serve as a sample domain grid to confirm an appropriate model function and to demonstrate the proof of concept. Unfortunately, all open MS data are provided in NIfTI format and the OpenMS data are the only data that come at least with the information on TE, TI, and TR and thus all sequence parameters needed in the simulation. In real-world applications, one can assume that manufacturers of models have access to the entire DICOM header that also includes tags for TE, TI, TR, and many more. Thus, in theory, more acquisition shifts caused by other sequence parameters could be incorporated as influencing factors in the stress tests. However, since the number of sampling points on the domain grid quickly rises with every additional influencing factor, a prior prioritization is crucial.

An intrinsic limitation of the T2w FLAIR and T1w sequences is that the CSF signal is very low or even nulled hampering partial volume estimation and relaxometry in this tissue. Accordingly, the differences between the simulations and measurements become most apparent in CSF compared to the other tissues, limiting the validation of the approach in CSF. Future work should investigate if tissue and relaxometry estimation can be improved by additionally incorporating the contrast of conventional T2w sequences in the first step of the image generation pipeline, as in these images CSF shows up brightly. All three scans (T2w, T2w FLAIR, and the post Gd T1w scan) constitute the “recommended core” in current MS scanning guidelines.^10^

Another limitation is the assumption that the average texture contribution to the signal is zero. This is not true in the case of artifacts resulting from inhomogeneities of B0, B1, or the receive coil sensitivity profile.^50^^,^^51^ The method is further only applicable to baseline images, of which the contrast can be fully described by the parameters accessible in the DICOM header; e.g., the parameter ${\mathrm{TE}}_{\text{last}}$ in Eq. (2) is approximated by 2·TE, since it is not part of the DICOM header. In the real volunteer scans, the true value for ${\mathrm{TE}}_{\text{last}}$ was 30% higher. In these experiments, changing the parameter to the correct value did not have any influence on the outcome of the comparison (due to the long TR value). Still, there might be other measures of contrast manipulation in T2w FLAIR studies that are not accessible by the DICOM tags and that prevent an accurate estimation of the DICOM scaling factor and thus the texture amplitude (e.g., modulated RF pulses to prevent the signal from decaying in long echo trains, acceleration techniques and dedicated k-space ordering, particularly common in 3D sequences,^25^^,^^52^^–^^55^ blood inflow,^56^ etc). Future work should elaborate to what extent these influences and their impact can be modeled and incorporated either in the simulation, e.g., by random guesses or in the stress tests represented by additional influencing factors.

Despite these limitations, the image simulation and stress test methodology presented in this work allows for investigation of the robustness of AI models in response to arbitrary data shifts. Due to the lack of a gold standard, the metrological proof of the F1 response to parameter changes is not possible and absolute predictions about these values remain uncertain. However, influencing parameters in the MR sequence can be compared with each other by the surface model coefficients and—given a tolerated performance drop—“safe” parameters settings can be at least roughly assessed (Fig. 4). Using the simulation algorithm as an alternative augmentation method also allows for introducing *a priori* knowledge on MR signal variations into the AI-model development process.

## Data Availability

The MS data utilized in this study are listed Table 2. The data policy of the clinical study does not allow free access to the volunteer MRI data. Due to the collaboration agreement with the industrial partner, the code cannot be made available.
